# Per-Capita Food Availability and Global Dementia Burden Across Multiple Exposure Windows

**DOI:** 10.3390/nu18162700

**Published:** 2026-08-18

**Authors:** Carmelo Mario Vicario, Simona Massimino, Vanni Caruso, Carmelo M. Porto, Enrico Nicosia, Alessio Avenanti, Antonino Pennisi, Alessandra Falzone, Sebastiano Nucera, Antonina Lax, Antonino Germanò, Francesca Ferraioli, Sabrina Castellano, Angelo Quartarone, Francesco Tomaiuolo

**Affiliations:** 1 Department of Cognitive Sciences, Psychology, Education and Cultural Studies, University of Messina, Via Concezione 6, 98121 Messina, Italy; simona.massimino@unime.it (S.M.);; 2 School of Pharmacy and Pharmacology, University of Tasmania, Sandy Bay, Hobart, TAS 7005, Australia; 3 Centro Studi e Ricerche in Neuroscienze Cognitive, Dipartimento di Psicologia “Renzo Canestrari”, Campus di Cesena, Università di Bologna, 47521 Cesena, Italy; 4 Centro de Investigación en Neuropsicología y Neurociencias Cognitivas, Universidad Católica del Maule, Talca 3460000, Chile; 5 Psychiatry Unit, Policlinico Universitario “Gaetano Barresi”, 98124 Messina, Italy; 6 Department of Clinical and Experimental Medicine, University Hospital of Messina, 98122 Messina, Italy; 7 Department of Educational Sciences, University of Catania, 95124 Catania, Italy; 8 IRCCS Centro Neurolesi Bonino-Pulejo, S.S. 113 Via Palermo, C.da Casazza, 98124 Messina, Italy

**Keywords:** dementia, food environment, nutritional exposome, environmental epidemiology, global health, nutrition transition

## Abstract

**Background/Objectives**: Per-capita food availability may represent a large-scale indicator of nutritional environments potentially associated with dementia risk. However, whether national dietary environments are associated with dementia burden across different exposure periods remains unclear. **Methods**: We linked model-derived estimates of per-capita food availability based on Food and Agriculture Organization (FAO) food supply data for 2005, 2010, and 2015 with 2018 age-standardized dementia incidence, prevalence, and mortality across 109 countries. Multivariable ordinary least-squares models were estimated with adjustment for socioeconomic, demographic, environmental, and metabolic covariates. **Results**: Higher per-capita food availability was positively associated with dementia outcomes in minimally adjusted models, particularly for the 2005 exposure window, although these associations were substantially attenuated after full covariate adjustment. In contrast, the 2010 exposure window showed positive associations with dementia incidence and prevalence after full adjustment using the conventional significance threshold (α = 0.05), whereas no significant associations were observed for mortality or for the 2015 exposure window. Sensitivity analyses indicated that associations varied according to exposure timing and model specification. **Conclusions**: Overall, these findings suggest that large-scale nutritional environments are associated with variations in dementia burden across countries, although the substantial temporal overlap among exposure measures precludes inference regarding temporally specific effects; these associations should therefore be interpreted within the broader socio-demographic and metabolic context in which national food environments are embedded.

## 1. Background

Chronic dietary abundance is a defining feature of the current global nutritional transition, paralleling global increases in obesity, metabolic syndrome, and type 2 diabetes [1,2]. Beyond metabolic diseases, nutritional excess is an emerging planetary health issue, given its links to food system pressures, resource use, and population aging. Although the long-term consequences of caloric excess on brain health remain incompletely understood, converging evidence from experimental, neuroimaging, and neuropathological studies suggests that sustained overnutrition is associated with systemic oxidative stress, insulin resistance, and microglial-mediated neuroinflammation, processes plausibly linked to neurodegeneration [3,4]. These findings provide biological context but do not imply that the ecological exposure examined in the present study reflects individual dietary intake.

Complementing these mechanistic models, recent population-based and autopsy studies have shown that individuals with obesity or chronic hyperglycemia exhibit greater cerebral microglial activation, accelerated cortical atrophy, and increased deposition of β-amyloid and phosphorylated tau relative to their normometabolic peers. Longitudinal analyses suggest that midlife caloric overconsumption is associated with a higher risk of dementia later in life [5,6]. More broadly, numerous epidemiological studies have linked obesity, metabolic dysfunction, and unhealthy dietary patterns with increased dementia risk at the individual level [7,8]. However, considerably less is known about whether characteristics of national food environments are associated with cross-country variation in dementia burden.

Previous ecological studies have explored the relationship between national dietary patterns and dementia risk, including analyses incorporating temporal lag structures between food supply and disease outcomes [9,10]. Nevertheless, evidence examining national per-capita food availability as a structural indicator of food-system characteristics remains limited. Accordingly, the extent to which such associations reflect independent effects of dietary environments, as opposed to broader socio-demographic and metabolic structures, remains unclear at the population level. Moreover, no prior study has examined national per-capita food availability as a structural correlate of cognitive outcomes embedded in food system transitions and planetary health frameworks.

The current phase of the global nutrition transition, characterized by excessive caloric availability and high consumption of ultra-processed foods, simultaneously contributes to environmental degradation and the global rise in noncommunicable diseases. Understanding how overnutrition relates to global food systems and health trajectories is essential for advancing the goals of planetary health and sustainable nutrition policies.

In the current study, we hypothesized that per-capita food availability, reflecting structural characteristics of national dietary environments, would be associated with variation in dementia burden across countries after accounting for key sociodemographic, environmental, and metabolic factors. To test this, we assembled a multi-country ecological dataset integrating per-capita dietary composition data from Zhao and You [11], who modelled national food availability patterns for 109 countries between 1990 and 2018. We extracted the years 2005, 2010, and 2015 to represent historical exposure windows and linked them to 2018 dementia outcomes (incidence, prevalence, and mortality) from the GBD [12] database of the Institute for Health Metrics and Evaluation (IHME).

Exposure values were drawn from the country-level dietary structure estimates reported by Zhao and You [11], derived from FAO food supply data, which describe national per-capita food availability across 109 developing and industrialized countries during 1990–2018. The present study extends prior work by coupling high-resolution global dietary structure data with longitudinal dementia outcomes, introducing a temporally structured ecological framework to examine associations across multiple exposure periods, an approach not previously applied to neurodegenerative risk at the population scale. Therefore, we examined whether per-capita food availability was associated with subsequent dementia burden, adjusting for sociodemographic, environmental, and metabolic covariates obtained from the World Bank and GBD datasets. Given the ecological design, the objective was to evaluate population-level associations rather than infer causal relationships between national food environments and dementia outcomes.

To address these questions, we estimated year-specific multivariable ordinary least-squares (OLS) models with heteroskedasticity-consistent errors to quantify exposure–outcome associations. Sequential covariate-adjustment models were used to evaluate the stability of these associations under increasing structural control.

To further examine the joint coefficient pattern across the highly correlated exposure windows, we estimated ridge-regularized distributed-lag models including the 2005, 2010, and 2015 food-availability measures simultaneously. Ridge regularization was used to reduce instability arising from the strong correlations between exposure windows. These analyses were intended as exploratory assessments of the distribution of coefficients across temporally overlapping exposure measures rather than as evidence of independent or temporally specific effects.

## 2. Methods

We integrated longitudinal data from 109 countries across all major world regions and conducted a country-level ecological analysis linking national per-capita food availability with age-standardized dementia incidence, prevalence, and mortality in 2018. Dementia outcomes were obtained from the Global Burden of Disease IHME-GBD [12] database and defined according to ICD-10 codes F00–F03 and G30–G31 [12].

Per-capita food availability data were drawn from Zhao and You [11], who modelled the dietary structure of 109 countries between 1990 and 2018 by harmonizing FAO food supply statistics into consistent country-level matrices. Specifically, we extracted country-level estimates of model-derived per-capita food availability (grams per person per day) across multiple food categories, as provided by Zhao and You [11] based on FAO food supply [1]. Dementia outcomes were evaluated for 2018, corresponding to the final year covered by the exposure dataset and allowing all historical exposure windows to be related to a common outcome reference. These variables represent the average daily availability of different food commodities at the national level, rather than observed intake. We interpret these estimates as a proxy for national dietary abundance because, at the ecological scale, aggregate food availability captures structural characteristics of the food system (e.g., overall caloric supply, dietary composition, and food system capacity), while not directly reflecting individual dietary intake. Accordingly, within this ecological framework, the exposure variable is interpreted as an indicator of the national food environment rather than as direct evidence of individual-level overconsumption.

We extracted the dietary structure values for 2005, 2010, and 2015 to represent exposures occurring approximately 13, 8, and 3 years before the 2018 dementia outcomes. Examining multiple historical exposure windows, rather than assuming a single exposure–outcome interval, allowed us to evaluate the consistency of ecological associations across different temporal intervals. Given the substantial temporal correlation expected among national food-availability estimates, these exposure windows were not considered independent or interpreted as representing distinct biological latency periods.

Although dietary structure estimates were available from 1990 onward, the present analysis was restricted to 2005–2015 to ensure consistent alignment between exposure data, age-standardized dementia outcomes, and the full set of socioeconomic, demographic, environmental, and metabolic covariates derived from the World Bank World Development Indicators (WDI) and GBD databases. Earlier years (1990–2000) exhibited substantially lower completeness and greater cross-national inconsistency for several key covariates, particularly for metabolic and environmental indicators. Inclusion of these periods would markedly reduce the effective sample size and introduce systematic selection of countries with more complete historical records, predominantly high-income settings. To preserve statistical power, cross-national comparability, and global representativeness, 2005 was selected as the earliest exposure window for fully adjusted models, reflecting a balance between temporal depth and harmonized data coverage across countries. Country-level covariates were obtained from two sources. Socio-demographic indicators—including gross domestic product (GDP) per capita, life expectancy, population aged ≥65 years, and urbanization—were extracted from the World Bank World Development Indicators (WDI). Health- and risk-related variables—including ambient air pollution, high alcohol use, high body mass index (BMI), high fasting plasma glucose, and smoking—were obtained from the IHME Global Burden Disease (GBD) database. These variables correspond to GBD rate estimates for the selected risk factors and therefore should not be interpreted as direct physical quantities (e.g., ambient PM_2.5_ concentration, litres of alcohol consumed, body mass index, fasting plasma glucose concentration, or smoking prevalence). Rather, they represent standardized epidemiological estimates derived within the GBD modelling framework.

Descriptive statistics for all covariates across study years are reported in Table S1. Covariates were aligned to each exposure year and analyses were restricted to countries with complete data (2005, n = 92; 2010, n = 105; 2015, n = 106).

###  Statistical Analysis

Year-specific multivariable ordinary least-squares (OLS) models were estimated separately for each exposure window according to the specification $Y_{j}=\beta X_{j,t}+\gamma^{⊤}Z_{j,t}+\varepsilon_{j}$, where $Y_{j}$ denotes the 2018 age-standardized dementia outcome in country j, $X_{j,t}$ represents per-capita food availability in exposure year t, $Z_{j,t}$ is the vector of covariates, and $\varepsilon_{j}$ is a country-level disturbance term. Because the design was cross-sectional (one outcome observation per country), serial autocorrelation over time was not assumed. HC3 heteroskedasticity-robust standard errors were applied to address cross-country heteroskedasticity and potential leverage effects in the moderate samples. The HC3 estimator provides a conservative small-sample correction and reduces the influence of high-leverage observations on the variance estimation. Standard regression diagnostics were examined to evaluate model adequacy, including assessment of multicollinearity using variance inflation factors (VIFs) and evaluation of potentially influential observations using leverage, Cook’s distance, and externally studentized residuals. Conventional diagnostic thresholds were defined as leverage > 2p/N, where p denotes the number of estimated regression parameters including the intercept, Cook’s D > 4/N, and |externally studentized residual| > 3. Residual behavior and potential departures from homoskedasticity were also examined. Diagnostic results are reported in Supplementary Table S14. Observations exceeding diagnostic thresholds were treated as potential influence flags rather than automatic criteria for exclusion; their impact was further evaluated through the leave-one-country-out sensitivity analysis described below. All variables were z-standardized within each exposure-year analytical sample to facilitate comparison of standardized regression coefficients across predictors and models. To evaluate the stability of associations under increasing covariate adjustment, sequential covariate-adjustment models were additionally estimated for each exposure year and dementia outcome. These models first included per-capita food availability alone and examined associations under a series of alternative covariate-adjustment specifications, culminating in the fully adjusted model. This procedure was implemented to assess the extent to which observed associations were sensitive to adjustment for correlated macro-level indicators.

As an additional sensitivity analysis, potential nonlinearity was evaluated by refitting each fully adjusted year-specific OLS model after including a quadratic term for standardized per-capita food availability. The statistical significance of the quadratic term and changes in model fit relative to the corresponding linear model were examined. Multicollinearity was assessed using variance inflation factors (VIFs). As is well established, multicollinearity primarily affects the precision of coefficient estimates by increasing standard errors rather than inflating statistical significance [13,14].

To further assess sensitivity to model specification, alternative covariate-adjustment sets were examined. These included a food-availability-only model; a structural model adjusted for GDP per capita, life expectancy, population aged ≥65 years, urbanization, and air pollution; a structural-plus-behavioral model additionally adjusted for high alcohol consumption and smoking; and the fully adjusted model, which further included high body mass index and high fasting plasma glucose. The same year-specific complete-case sample was retained across these specifications to distinguish changes associated with covariate adjustment from those due to changes in sample composition. These models were used as a sensitivity analysis of progressively conditioned ecological associations and were not intended as formal mediation models. Results are reported in Supplementary Table S11.

A leave-one-country-out (LOCO) sensitivity analysis was also conducted for each fully adjusted year-specific model. Each country was excluded in turn, all variables were re-standardized within the reduced sample, and the model was re-estimated using HC3 robust standard errors. The resulting range of food-availability coefficients and *p*-values was examined to assess whether the year-specific associations were disproportionately influenced by any individual country (Supplementary Table S12).

To explore temporal overlap between exposure windows, a ridge distributed-lag model was estimated according to the specification $Y_{j}=\beta_{2005}X_{j,2005}+\beta_{2010}X_{j,2010}+\beta_{2015}X_{j,2015}+\gamma^{⊤}Z_{j}+\varepsilon_{j}$. Because the three exposure windows were highly correlated, ridge regularization (L2 penalty) was applied as an exploratory approach to stabilize coefficient estimation under multicollinearity. All predictors and outcomes were z-standardized prior to model estimation. The ridge penalty parameter (λ) was selected using 10-fold cross-validation over a logarithmically spaced grid of 161 candidate values ranging from $10^{-4}$ to $10^{4}$. Given the exploratory purpose of this analysis, the ridge model was intended to complement, rather than replace, the primary year-specific OLS analyses. The model was used to examine the distribution of coefficients across the highly correlated exposure windows without interpreting them as independent or temporally specific effects. Uncertainty intervals for ridge coefficients were estimated using non-parametric bootstrap resampling (1000 replicates), with λ fixed at the value selected in the original cross-validation procedure. To assess sensitivity to the degree of regularization, each ridge model was additionally re-estimated using penalty parameters corresponding to 0.1× and 10× the cross-validated λ. This analysis was used to evaluate whether the relative coefficient pattern across the correlated exposure windows depended on the selected regularization strength (Supplementary Table S13).

Exposure-year effects refer to the standardized regression coefficients obtained from the year-specific models. For each exposure year, coefficients across dementia outcomes were summarized using inverse-variance weighting, where each coefficient was weighted by the inverse of its squared robust standard error. This procedure was used as a descriptive synthesis of effect sizes across related but conceptually distinct outcomes (incidence, prevalence, and mortality), rather than as a formal meta-analytic inference. Each outcome was analysed separately, and pooled estimates were used only to summarise the overall consistency and magnitude of associations across exposure windows.

To ensure comparability across exposure windows, a balanced-sample sensitivity analysis was additionally conducted by restricting analyses to countries with complete data across all exposure years and covariates (n = 92). Correlations between exposure measures across years were also examined to quantify temporal overlap between food-availability estimates. Two-sided tests used *p* < 0.05 as the primary significance threshold. Given nine primary exposure–outcome tests (three exposure years × three dementia outcomes), a conservative Bonferroni-adjusted threshold (α = 0.0056) was additionally considered in sensitivity analyses. All analyses were conducted in Python (version 3.12) using statsmodels 0.14. All data were aggregated and publicly available.

## 3. Results

**Figure 1:**
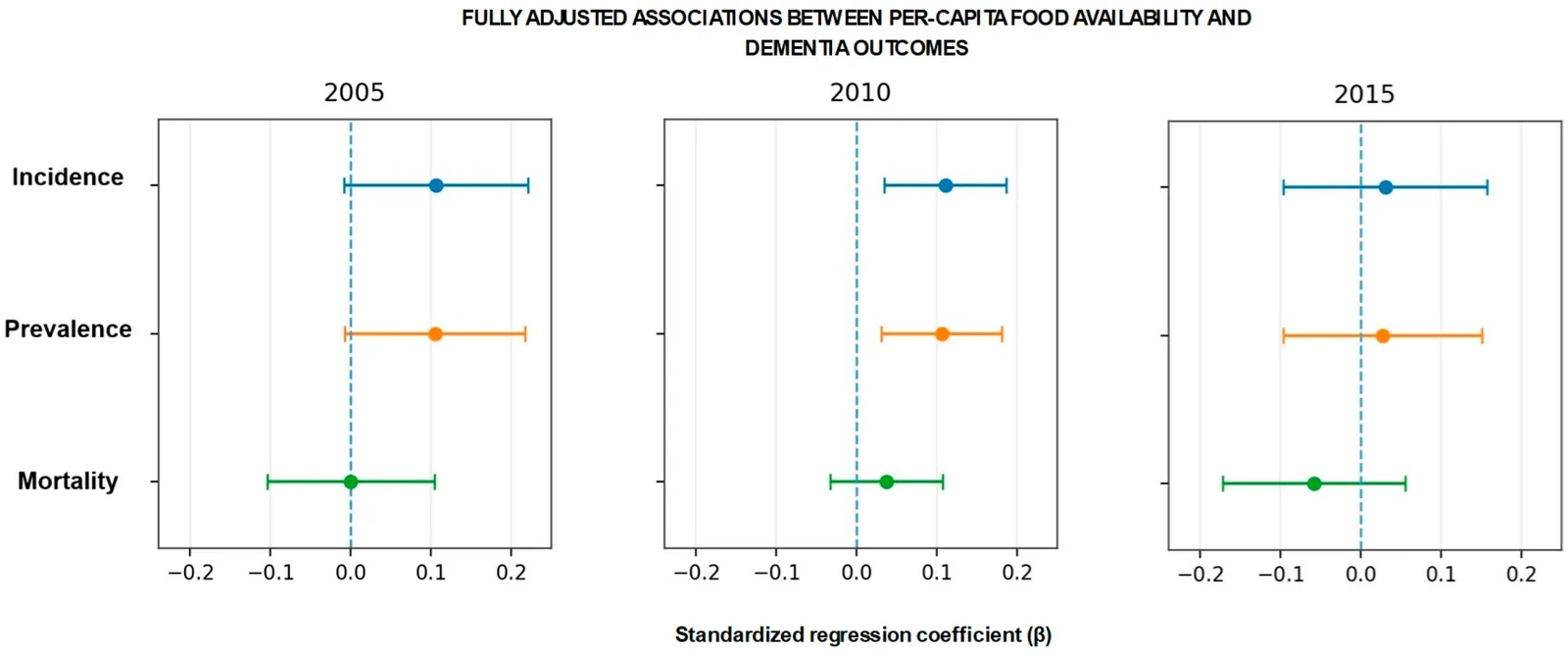
Fully adjusted associations between per-capita food availability and 2018 dementia outcomes across three historical exposure windows. Panels show standardized regression coefficients (β) and 95% confidence intervals (derived from HC3 heteroskedasticity-robust standard errors) from multivariable ordinary least-squares (OLS) models for dementia incidence, prevalence, and mortality using year-specific per-capita food availability data from 2005, 2010, and 2015, respectively. Models were adjusted for the full set of socio-demographic and health-related covariates listed in Table S1. The dashed vertical line indicates the null association (β = 0).

Descriptive statistics for country-level covariates across exposure years are reported in Supplementary Table S1. Year-specific analyses showed that per-capita food availability in 2005 (n = 92 countries) was positively associated with 2018 dementia outcomes in minimally adjusted models, but these associations were substantially attenuated after inclusion of the full covariate set for incidence (β = 0.106, *p* = 0.071), prevalence (β = 0.105, *p* = 0.067), and mortality (β = −0.000, *p* = 0.999) (Table S2). Across exposure windows, the 2010 models (n = 105) showed positive associations with dementia incidence (β = 0.110, *p* = 0.004) and prevalence (β = 0.106, *p* = 0.006), whereas no significant association was observed for mortality (β = 0.037, *p* = 0.303) (Table S3; Figure 1). Both incidence and prevalence reached statistical significance at the conventional α = 0.05 threshold; however, only the association with dementia incidence remained significant after Bonferroni correction for multiple comparisons (α = 0.0056).

For 2015 (n = 106), associations were consistently null across all outcomes (incidence: β = 0.030, *p* = 0.638; prevalence: β = 0.027, *p* = 0.670; mortality: β = −0.059, *p* = 0.311; Table S4).

To further assess the robustness of these findings, sequential covariate-adjustment models indicated that associations were sensitive to covariate inclusion and were substantially attenuated after full adjustment (Supplementary Table S5). For example, the association between 2005 per-capita food availability and dementia incidence decreased from β = 0.722 in the unadjusted model to β = 0.106 after full adjustment, with a similar attenuation observed for dementia prevalence. Multicollinearity diagnostics (VIFs) are reported in Supplementary Table S6. Moderate collinearity was observed for several metabolic and environmental covariates, particularly in the 2005 and 2010 models, whereas generally lower VIF values were observed in the 2015 model. The variance inflation factor for per-capita food availability remained low across all exposure years. Additional regression diagnostics identified some high-leverage and potentially influential country observations across the fully adjusted models, with some observations exceeding conventional thresholds for leverage, Cook’s distance, or externally studentized residuals (Supplementary Table S14). These diagnostic flags were not used as automatic exclusion criteria. Importantly, the complementary leave-one-country-out analyses indicated that the principal findings were not driven by any single country (Supplementary Table S12). Additional sensitivity analyses evaluating the linearity assumption by including a quadratic term for standardized per-capita food availability in each fully adjusted year-specific model yielded no statistically significant quadratic effects (all *p* ≥ 0.155), Supplementary Table S7), providing no evidence of a quadratic association within the examined specifications.

To ensure comparability across exposure windows, we conducted a balanced-sample sensitivity analysis restricted to countries with complete data across all three exposure years and covariates (n = 92). The results were broadly consistent with the main analyses across outcomes, with associations generally attenuated after full covariate adjustment (Supplementary Table S8). In the same balanced sample, exposure measures were strongly correlated across time (2005–2010: r = 0.866, *p* < 0.001; 2010–2015: r = 0.964, *p* < 0.001; 2005–2015: r = 0.828, *p* < 0.001; Supplementary Table S9), indicating substantial temporal overlap between exposure windows. Given this high temporal correlation, the exploratory ridge distributed-lag analysis was interpreted descriptively and did not provide a basis for distinguishing independent or temporally specific exposure effects. Sensitivity analyses using alternative regularization strengths (0.1× and 10× the cross-validated λ) showed some variation in the relative magnitude of exposure-year coefficients, particularly for incidence and prevalence, whereas mortality coefficients were essentially unchanged. At the cross-validated λ, all 95% bootstrap uncertainty intervals included zero (Supplementary Table S13), further supporting a cautious, descriptive interpretation of the ridge coefficients.

The list of countries included in the balanced sample is reported in Supplementary Table S10. Inverse-variance pooling of standardized coefficients across incidence, prevalence, and mortality outcomes yielded pooled estimates of β_(2005)_ = 0.066, β_(2010)_ = 0.082, and β_(2015)_ = −0.004. These pooled estimates were used only as a descriptive summary across outcomes and should not be interpreted as evidence of differential temporal effects.

Additional analyses using alternative covariate-adjustment sets showed that the marked attenuation of the 2005 associations was already evident after adjustment for structural country-level characteristics, before the inclusion of BMI and fasting plasma glucose. The 2010 specification showed comparatively greater consistency across alternative adjustment sets, particularly for dementia incidence, whereas the 2015 associations remained weak after multivariable adjustment (Supplementary Table S11). Leave-one-country-out analyses further indicated that the 2010 incidence and prevalence associations were not driven by any single country: both remained significant at the conventional α = 0.05 threshold in all 105 country-deletion iterations. The incidence association remained below the Bonferroni-adjusted threshold (α = 0.0056) in 87/105 iterations, compared with 25/105 iterations for prevalence. In contrast, the 2015 associations remained non-significant in all iterations, while the 2005 incidence and prevalence associations reached nominal significance in only 4/92 iterations (Supplementary Table S12).

## 4. Discussion

This study provides global ecological evidence that per-capita food availability, as an indicator of structural dietary environments, is associated with cross-national variation in dementia burden, although these associations are highly sensitive to covariate adjustment. These findings should be interpreted as country-level ecological associations and not as evidence of causal effects at the individual level.

In minimally adjusted models, higher food availability was associated with greater dementia incidence, prevalence, and mortality, particularly for the 2005 exposure window. However, these associations were substantially attenuated and no longer statistically significant after full adjustment for socioeconomic, environmental, and metabolic factors. Importantly, although dementia outcomes were age-standardized, the fully adjusted models also accounted for population ageing to address residual cross-national differences in demographic structure beyond age standardization alone. In contrast, for the 2010 exposure window, positive associations with dementia incidence and prevalence remained evident after full covariate adjustment. These associations reached statistical significance at the conventional α = 0.05 threshold, although only the association with dementia incidence remained significant after Bonferroni correction for multiple comparisons. No significant associations were observed for mortality, and no associations were detected for the 2015 exposure window.

This pattern suggests that the observed associations varied across exposure windows, with the 2010 specification showing the most consistent associations, particularly for dementia incidence, although the substantial temporal overlap among exposure windows precludes inference regarding a distinct or optimal exposure period.

This pattern was further examined using exploratory ridge distributed-lag analyses, which were used to assess coefficient patterns across the highly correlated exposure windows. However, these findings should be interpreted cautiously given the substantial temporal overlap among exposure windows and the exploratory role of the ridge analysis, which was intended to complement rather than replace the primary year-specific OLS models. Sensitivity analyses across alternative regularization strengths showed some variation in the relative magnitude of exposure-year coefficients, particularly for incidence and prevalence, and all bootstrap uncertainty intervals at the cross-validated λ included zero. Accordingly, the ridge coefficients should be interpreted descriptively and cannot be interpreted as independent or temporally specific exposure effects.

The absence of statistically significant associations after full adjustment for the 2005 exposure window provides limited support for a simple cumulative or long-lag pattern, although this interpretation should be considered in light of the substantial correlations among exposure windows.

The nominally more consistent associations observed for the 2010 specification should therefore not be interpreted as evidence of an intermediate temporal alignment between exposure and outcome. Previous evidence suggests that associations with dementia risk may depend on exposure timing, with intermediate life-course measures sometimes showing stronger relationships than those assessed more proximally or distally from disease onset [5,15]. However, the high correlations among exposure windows in the present study prevent attribution of the observed pattern to a specific biological latency period.

Results from the balanced-sample sensitivity analysis were consistent with the main findings, suggesting that these patterns were not driven by differences in country composition. At the same time, strong correlations between exposure measures across years indicate substantial temporal overlap between exposure windows and suggest that per-capita food availability primarily reflects relatively stable structural characteristics of national food systems. Accordingly, the three exposure windows should not be interpreted as independent temporal exposures but rather as overlapping representations of relatively stable national dietary environments, limiting the attribution of window-specific effects.

Additional sensitivity analyses further supported the robustness of the main findings while clarifying the role of covariate adjustment. Alternative covariate specifications showed that the marked attenuation of the 2005 associations was already evident after adjustment for structural country-level characteristics, before BMI and fasting plasma glucose were introduced. Regression diagnostics identified some high-leverage and potentially influential country-level observations; however, leave-one-country-out analyses further indicated that the nominal 2010 incidence and prevalence associations were not driven by any single country, although only the incidence association showed substantial robustness to the Bonferroni-adjusted threshold. Overall, these findings suggest that ecological associations between food availability and dementia burden are embedded within broader socio-demographic and macro-structural systems shaping population-level brain health. These findings are broadly consistent with previous ecological studies reporting associations between national dietary environments and dementia burden after considering delayed exposure–outcome relationships [9,10]. However, unlike these earlier studies, the present analysis evaluated multiple historical exposure windows while accounting for a broad set of socioeconomic, environmental, and metabolic covariates, demonstrating that the observed associations were highly sensitive to structural adjustment and not uniformly present across exposure periods.

A substantial body of literature supports the biological plausibility of links between dietary environments and neurodegenerative processes. Sustained energy imbalance has been associated with insulin resistance, endothelial dysfunction, oxidative stress, and chronic neuroinflammation, all of which are implicated in dementia pathophysiology [16,17,18,19,20]. Observational studies further indicate that obesity, metabolic dysfunction, and long-term exposure to environmental risk factors such as air pollution are associated with adverse neurocognitive outcomes [21,22,23,24]. Although these experimental and clinical findings provide biological plausibility for potential links between dietary environments and neurodegeneration, the present ecological analyses do not allow inference that the observed country-level associations are mediated through these mechanisms.

Neuroimaging and neuropathological studies provide convergent evidence linking metabolic dysfunction to neurodegenerative processes. Individuals with obesity or type 2 diabetes exhibit increased microglial activation, cerebral hypometabolism, and accelerated cortical thinning, while post-mortem analyses reveal greater amyloid-β and tau pathology in metabolically compromised individuals [25,26,27,28]. These findings suggest that chronic metabolic disturbances may act as long-term neurobiological stressors. Although such mechanisms cannot be directly tested in the present study, they offer a plausible framework for understanding how large-scale dietary environments may relate to population-level dementia burden.

From a global health perspective, regions with higher per-capita food availability—such as Western Europe, East Asia, North America, and Australia—largely correspond to high-income settings characterized by widespread availability of energy-dense and highly processed foods [29,30,31]. This geographical correspondence is consistent with a broader feature of modern food systems, in which affluence coexists with dietary environments marked by high caloric supply and increasing reliance on ultra-processed products.

As these conditions extend to middle-income countries undergoing nutritional transition, even modest shifts in population-level risk factors may translate into substantial variation in dementia burden. However, unmeasured contextual factors—including healthcare access, diagnostic capacity, registry completeness, reporting practices, survival following dementia diagnosis, and competing mortality—are also likely to contribute, and per-capita food availability may therefore capture broader processes of socioeconomic development and health-system capacity rather than an isolated nutritional exposure. Importantly, as an ecological indicator derived from national food balance sheets, per-capita food availability reflects the overall structure of national food environments rather than actual food consumption by individuals. Consequently, countries with similar average food availability may differ substantially in food distribution, socioeconomic inequalities, and patterns of overnutrition, factors that cannot be captured by aggregate national estimates.

This study has several limitations. The ecological design precludes inference at the individual level, and per-capita food availability represents a model-derived indicator of national food supply rather than a direct measure of dietary intake. Accordingly, the present findings should not be interpreted as evidence that increasing food availability causes dementia risk. Rather, per-capita food availability should be viewed as a structural ecological indicator that may capture multiple interrelated characteristics of national food systems, socioeconomic development, and population health.

Within-country heterogeneity and qualitative aspects of diet, such as nutrient composition and food processing, are not captured. Likewise, the measure does not account for inequalities in food distribution or access within countries, which may substantially influence individual nutritional exposure despite similar levels of average food availability. Cross-national indicators may also vary in completeness and accuracy, particularly in low- and middle-income settings, potentially introducing measurement error and attenuation bias. Residual confounding by unmeasured structural and lifestyle factors—including healthcare systems, healthcare access, diagnostic capacity, educational attainment, cultural dietary patterns, physical activity, and environmental co-exposures—remains possible despite extensive covariate adjustment.

Some covariates included in the fully adjusted models, particularly body mass index and fasting plasma glucose, may plausibly function both as confounders and as downstream metabolic intermediates linking national food environments to dementia burden.

However, the alternative covariate-set sensitivity analysis showed that the marked attenuation of the 2005 associations was already evident after adjustment for structural country-level characteristics, before BMI and fasting plasma glucose were introduced. Thus, although some degree of over-adjustment through metabolic factors cannot be excluded, it does not appear to fully account for the attenuation observed in the fully adjusted models. Accordingly, the structural and fully adjusted specifications should not be interpreted as progressively more conservative estimates of the same underlying effect, because they condition on different sets of country-level characteristics. More generally, the alternative adjustment specifications should be viewed as estimating different conditional ecological associations rather than as identifying direct and mediated causal effects. Finally, the use of a single outcome year precluded assessment of the temporal stability of these ecological associations across successive dementia estimates. Future studies incorporating multiple outcome years may help determine whether the observed patterns remain consistent over time.

In conclusion, per-capita food availability is associated with dementia burden in cross-national ecological analyses, but these associations are highly sensitive to full structural adjustment and model specification. The 2010 specification showed nominally more consistent associations, particularly for dementia incidence, but the strong correlation among exposure windows precludes interpretation of this pattern as evidence of a distinct temporal effect. These findings support the interpretation that per-capita food availability represents a structural ecological correlate of dementia burden rather than evidence of an independent causal nutritional determinant.

Future longitudinal and biomarker-based studies will be needed to clarify the extent to which long-term nutritional environments independently contribute to dementia risk and to disentangle their interaction with vascular, metabolic, and inflammatory pathways.

## Data Availability

All data used in this study are publicly available and can be accessed from the Institute for Health Metrics and Evaluation (IHME) Global Burden of Disease (https://ghdx.healthdata.org/gbd-results-tool), World Bank Open Data portal (https://data.worldbank.org), and Zhao and You (2024) [11] (*Environ. Sci. Technol.*, 58, 8709–8723, https://pubs.acs.org/doi/10.1021/acs.est.4c00010).

## Supplementary Materials

The following supporting information can be downloaded at https://www.mdpi.com/article/10.3390/nu18162700/s1.
