# Supplementary material for: Per-Capita Food Availability and Global Dementia Burden Across Multiple Exposure Windows
Source: Nutrients. 2026 Aug 18;18(16):2700. doi: 10.3390/nu18162700 (PMC13516366; doi:10.3390/nu18162700)
Supplement: Supplementary file 1 [file nutrients-18-02700-s001.zip › nutrients-4407705-supplementary.pdf]

Supplementary Tables

Table S1. Descriptive statistics of country-level covariates across exposure years

Variable	2005 Mean	2005 SD	2010 Mean	2010 SD	2015 Mean	2015 SD
GDP per capita (US$)	10,772	16,536	14,000	20,768	13,854	20,265
Life expectancy (years)	67.08	10.46	69.62	9.40	71.10	8.52
Population ≥65 (total)	4,163,942	12,803,950	4,524,718	13,747,350	5,125,513	16,095,950
Urbanisation (%)	53.49	23.03	55.66	22.88	57.54	22.62
Air pollution (GBD Rate)	5.17	4.33	5.80	4.51	6.81	3.19
High alcohol use (GBD Rate)	−0.20	0.25	−0.23	0.27	−0.26	0.30
High body-mass index (GBD Rate)	0.98	1.30	1.26	1.49	1.54	1.81
High fasting plasma glucose (GBD Rate)	2.87	3.17	3.40	3.53	4.02	4.20
Smoking (GBD Rate)	0.56	0.66	0.63	0.67	0.66	0.70

Note. Socio-demographic variables were obtained from the World Bank World Development Indicators (WDI). Health- and risk-related variables were obtained from the Global Burden of Disease (IHME-GBD) database. The five IHME-GBD covariates (air pollution, high alcohol use, high body-mass index, high fasting plasma glucose, and smoking) correspond to GBD rate estimates for the selected risk factors and therefore should not be interpreted as direct physical quantities (e.g., μg/m³, litres per capita, kg/m², mmol/L, or prevalence percentages). These variables are reported in their original extracted metric for descriptive purposes. All covariates were z-standardized within each exposure year prior to regression analyses.

Table S2. Year-specific OLS (2005 exposure), all predictors

Predictor	Incidence β (SE)	Incidence *p*	Prevalence β (SE)	Prevalence *p*	Deaths β (SE)	Deaths *p*
Food availability	0.106 (0.059)	0.071	0.105 (0.057)	0.067	−0.000 (0.053)	0.999
GDP per capita	−0.110 (0.111)	0.320	−0.116 (0.108)	0.283	−0.021 (0.102)	0.839
Life expectancy	0.163 (0.105)	0.120	0.169 (0.102)	0.098	0.101 (0.093)	0.276
Population ≥65 (total)	0.053 (0.098)	0.590	0.054 (0.095)	0.569	0.004 (0.087)	0.964
Urbanisation	−0.015 (0.090)	0.866	−0.018 (0.087)	0.833	−0.017 (0.084)	0.842
Air pollution	0.096 (0.104)	0.355	0.081 (0.102)	0.425	0.270 (0.099)	0.006
High alcohol use	−0.064 (0.092)	0.484	−0.051 (0.089)	0.567	−0.046 (0.083)	0.580
High body-mass index	0.081 (0.094)	0.392	0.083 (0.091)	0.359	−0.069 (0.085)	0.419
High fasting plasma glucose	0.622 (0.113)	<0.001	0.628 (0.109)	<0.001	0.661 (0.101)	<0.001
Smoking	0.015 (0.094)	0.873	0.031 (0.091)	0.732	0.049 (0.084)	0.563

Note. Year-specific multivariable ordinary least-squares (OLS) models were estimated for the 2005 exposure window (**N = 92**). All variables were z-standardized (z-scores) within the 2005 analytical sample prior to analysis; therefore, regression coefficients (β) represent standardized effect sizes that are directly comparable across predictors. Heteroskedasticity-robust standard errors (**HC3**) were used to account for potential heteroskedasticity and influential observations. Models included per-capita food availability as the primary exposure and were simultaneously adjusted for gross domestic product (GDP) per capita, life expectancy, population aged ≥65 years (total), urbanisation, air pollution, high alcohol use, high body-mass index, high fasting plasma glucose, and smoking. Health-related covariates were obtained from the Global Burden of Disease (IHME-GBD) database and correspond to **modelled population-level rate estimates**. Values in parentheses denote HC3 robust standard errors.

Table S3_extended — Year-specific OLS (2010 exposure), all predictors

Predictor	Incidence β (SE)	Incidence *p*	Prevalence β (SE)	Prevalence *p*	Deaths β (SE)	Deaths *p*
Food availability	0.110 (0.039)	0.004	0.106 (0.038)	0.006	0.037 (0.036)	0.303
GDP per capita	−0.031 (0.049)	0.523	−0.038 (0.054)	0.490	0.020 (0.051)	0.695
Life expectancy	0.133 (0.050)	0.008	0.139 (0.051)	0.006	0.055 (0.038)	0.151
Population ≥65 (total)	0.036 (0.046)	0.434	0.037 (0.049)	0.457	0.001 (0.032)	0.973
Urbanisation	−0.042 (0.043)	0.325	−0.045 (0.043)	0.302	−0.033 (0.050)	0.503
Air pollution	0.187 (0.103)	0.068	0.176 (0.111)	0.112	0.260 (0.082)	0.002
High alcohol use	−0.022 (0.073)	0.763	−0.012 (0.075)	0.873	−0.019 (0.054)	0.727
High body-mass index	0.128 (0.179)	0.475	0.128 (0.178)	0.473	0.006 (0.266)	0.982
High fasting plasma glucose	0.535 (0.189)	0.005	0.539 (0.193)	0.005	0.622 (0.298)	0.037
Smoking	0.006 (0.108)	0.956	0.024 (0.118)	0.836	0.061 (0.077)	0.431

Note. Year-specific multivariable ordinary least-squares (OLS) models were estimated for the 2010 exposure window (**N = 105**) using the complete-case sample. All variables were z-standardized (z-scores) within the analytical sample prior to analysis; therefore, regression coefficients (β) represent standardized effect sizes. Heteroskedasticity-robust standard errors (**HC3**) were used throughout. Models included per-capita food availability as the primary exposure together with all covariates listed in **Table S1**. Values in parentheses denote HC3 robust standard errors.

Table S4_extended — Year-specific OLS (2015 exposure), all predictors

Predictor	Incidence β (SE)	Incidence *p*	Prevalence β (SE)	Prevalence *p*	Deaths β (SE)	Deaths *p*
**Food availability**	0.030 (0.065)	0.638	0.027 (0.063)	0.670	−0.059 (0.058)	0.311
**GDP per capita**	−0.055 (0.047)	0.243	−0.061 (0.048)	0.199	−0.008 (0.029)	0.777
**Life expectancy**	0.090 (0.047)	0.054	0.096 (0.047)	0.041	0.023 (0.036)	0.516
**Population ≥65 (total)**	0.038 (0.078)	0.622	0.038 (0.079)	0.626	0.002 (0.021)	0.919
**Urbanisation**	−0.006 (0.046)	0.898	−0.008 (0.046)	0.860	0.004 (0.047)	0.935
**Air pollution**	−0.075 (0.028)	0.008	−0.076 (0.029)	0.009	−0.016 (0.021)	0.452
**High alcohol use**	−0.021 (0.072)	0.775	−0.009 (0.074)	0.907	−0.044 (0.041)	0.286
**High body-mass index**	0.165 (0.123)	0.178	0.163 (0.118)	0.168	0.060 (0.132)	0.650
**High fasting plasma glucose**	0.620 (0.104)	<0.001	0.613 (0.104)	<0.001	0.759 (0.163)	<0.001
**Smoking**	0.123 (0.109)	0.256	0.146 (0.117)	0.213	0.162 (0.052)	0.002

Note. Year-specific multivariable ordinary least-squares (OLS) models were estimated for the 2015 exposure window (N = 106) using the complete-case sample. All variables were z-standardized (z-scores) within the analytical sample prior to analysis; therefore, regression coefficients (β) represent standardized effect sizes. Heteroskedasticity-robust standard errors (HC3) were used throughout. Models included per-capita food availability as the primary exposure together with all covariates listed in Table S1. Values in parentheses denote HC3 robust standard errors.

Table S5

Table S5A. Covariate-adjustment sensitivity models — Incidence rate

Balanced sample, N = 92

Outcome	Exposure year	Model	β (Food)	SE	95% CI	p-value
Incidence	2005	FAO only	0.722	0.088	[0.550, 0.895]	<0.001
		+ GDP	0.436	0.120	[0.201, 0.670]	<0.001
		+ Life expectancy	0.367	0.123	[0.127, 0.607]	0.003
		+ Air pollution	0.295	0.093	[0.113, 0.477]	0.001
		+ High BMI	0.222	0.090	[0.045, 0.399]	0.014
		+ High fasting glucose	0.185	0.060	[0.067, 0.302]	0.002
		Full model	0.106	0.059	[−0.009, 0.221]	0.071
	2010	FAO only	0.661	0.076	[0.511, 0.811]	<0.001
		+ GDP	0.361	0.101	[0.162, 0.560]	<0.001
		+ Life expectancy	0.292	0.114	[0.070, 0.515]	0.010
		+ Air pollution	0.273	0.083	[0.110, 0.435]	0.001
		+ High BMI	0.133	0.089	[−0.040, 0.307]	0.132
		+ High fasting glucose	0.179	0.044	[0.093, 0.266]	<0.001
		Full model	0.093	0.042	[0.010, 0.176]	0.028

Outcome	Exposure year	Model	β (Food)	SE	95% CI	p-value
	2015	FAO only	0.617	0.084	[0.453, 0.782]	<0.001
		+ GDP	0.341	0.093	[0.159, 0.522]	<0.001
		+ Life expectancy	0.219	0.111	[0.002, 0.436]	0.048
		+ Air pollution	0.601	0.081	[0.442, 0.760]	<0.001
		+ High BMI	0.126	0.080	[−0.030, 0.282]	0.114
		+ High fasting glucose	0.164	0.050	[0.066, 0.262]	0.001
		Full model	0.065	0.043	[−0.019, 0.149]	0.129

Table S5B. Covariate-adjustment sensitivity models — Prevalence rate

Balanced sample, N = 92

Outcome	Exposure year	Model	β (Food)	SE	95% CI	p-value
Prevalence	2005	FAO only	0.721	0.088	[0.549, 0.893]	<0.001
		+ GDP	0.436	0.119	[0.203, 0.669]	<0.001
		+ Life expectancy	0.363	0.121	[0.126, 0.600]	0.003
		+ Air pollution	0.298	0.094	[0.114, 0.482]	0.002
		+ High BMI	0.223	0.088	[0.049, 0.396]	0.012
		+ High fasting glucose	0.186	0.060	[0.068, 0.304]	0.002
		Full model	0.105	0.057	[−0.007, 0.218]	0.067

Outcome	Exposure year	Model	β (Food)	SE	95% CI	p-value
	2010	FAO only	0.660	0.077	[0.510, 0.811]	<0.001
		+ GDP	0.361	0.101	[0.162, 0.559]	<0.001
		+ Life expectancy	0.290	0.113	[0.068, 0.512]	0.010
		+ Air pollution	0.275	0.085	[0.108, 0.442]	0.001
		+ High BMI	0.136	0.088	[−0.037, 0.309]	0.124
		+ High fasting glucose	0.181	0.045	[0.093, 0.270]	<0.001
		Full model	0.094	0.043	[0.010, 0.178]	0.028
	2015	FAO only	0.616	0.084	[0.452, 0.781]	<0.001
		+ GDP	0.340	0.092	[0.159, 0.521]	<0.001
		+ Life expectancy	0.217	0.110	[0.001, 0.432]	0.049
		+ Air pollution	0.600	0.081	[0.442, 0.759]	<0.001
		+ High BMI	0.127	0.079	[−0.028, 0.282]	0.109
		+ High fasting glucose	0.165	0.050	[0.067, 0.263]	<0.001
		Full model	0.063	0.043	[−0.022, 0.149]	0.144

Table S5C. Covariate-adjustment sensitivity models — Mortality rate

Balanced sample, N = 92

Outcome	Exposure year	Model	β (Food)	SE	95% CI	p-value
Mortality	2005	FAO only	0.663	0.096	[0.475, 0.850]	<0.001
		+ GDP	0.308	0.129	[0.056, 0.561]	0.017
		+ Life expectancy	0.304	0.145	[0.019, 0.589]	0.036
		+ Air pollution	0.151	0.098	[−0.041, 0.343]	0.123
		+ High BMI	0.108	0.070	[−0.029, 0.245]	0.121
		+ High fasting glucose	0.044	0.073	[−0.098, 0.187]	0.542
		Full model	−0.000	0.053	[−0.104, 0.104]	0.999
	2010	FAO only	0.608	0.077	[0.458, 0.758]	<0.001
		+ GDP	0.274	0.108	[0.062, 0.486]	0.011
		+ Life expectancy	0.241	0.127	[−0.009, 0.490]	0.058
		+ Air pollution	0.176	0.090	[−0.000, 0.353]	0.051
		+ High BMI	0.054	0.074	[−0.091, 0.199]	0.465
		+ High fasting glucose	0.077	0.039	[−0.001, 0.154]	0.052
		Full model	0.036	0.043	[−0.049, 0.121]	0.406
	2015	FAO only	0.557	0.083	[0.395, 0.718]	<0.001

Outcome	Exposure year	Model	β (Food)	SE	95% CI	p-value
		+ GDP	0.258	0.096	[0.069, 0.447]	0.007
		+ Life expectancy	0.148	0.128	[−0.102, 0.399]	0.246
		+ Air pollution	0.543	0.080	[0.387, 0.700]	<0.001
		+ High BMI	0.044	0.066	[−0.086, 0.174]	0.505
		+ High fasting glucose	0.057	0.020	[0.018, 0.096]	0.004
		Full model	−0.004	0.028	[−0.059, 0.050]	0.878

Notes. Sequential covariate-adjustment models were estimated using the balanced sample of countries with complete data across all exposure years and covariates (N = 92). The reported specifications include: (i) an unadjusted model containing per-capita food availability only; (ii) models containing food availability and each displayed covariate separately; and (iii) a fully adjusted model containing food availability and the complete covariate set listed in Table S1. All variables included in each model were z-standardized within the corresponding exposure year. Heteroskedasticity-robust standard errors (HC3) were used throughout. Coefficients represent standardized estimates for per-capita food availability under each adjustment specification.

Table S6. Multicollinearity diagnostics across exposure years

Year	Variable	VIF
**2005**	Food availability	2.79
	GDP per capita	3.89
	Life expectancy	3.34
	Population ≥65 (total)	1.20
	Urbanisation	2.33
	Air pollution	7.26
	High alcohol use	2.20
	High body-mass index	6.10
	High fasting plasma glucose	8.30
	Smoking	5.18
**2010**	Food availability	2.34
	GDP per capita	2.53
	Life expectancy	2.87
	Population ≥65 (total)	1.23
	Urbanisation	2.11
	Air pollution	4.98
	High alcohol use	2.46

	High body-mass index	5.19
	High fasting plasma glucose	7.06
	Smoking	5.09
**2015**	Food availability	2.20
	GDP per capita	2.42
	Life expectancy	3.17
	Population ≥65 (total)	1.24
	Urbanisation	1.91
	Air pollution	1.19
	High alcohol use	2.45
	High body-mass index	4.52
	High fasting plasma glucose	4.70
	Smoking	5.36

Notes. Variance Inflation Factors (VIFs) were computed for all predictors included in the fully adjusted year-specific regression models. All predictors were standardized (z-scores) within each exposure year prior to analysis. VIF values quantify the degree of multicollinearity among predictors. Moderate collinearity was observed for several metabolic and environmental covariates, particularly in the 2005 and 2010 models, whereas lower VIF values were generally observed in the 2015 model. The primary exposure (per-capita food availability) consistently showed low-to-moderate VIF values across all exposure years.

Table S7. Sensitivity analysis evaluating the linearity assumption using quadratic terms for standardized per-capita food availability

Standardized OLS regressions with heteroskedasticity-robust (HC3) standard errors.

Outcome	Exposure year	Quadratic term β	HC3 SE	95% CI	p-value
Incidence	2005	0.016	0.069	[−0.119, 0.150]	0.819
	2010	0.040	0.035	[−0.027, 0.108]	0.243
	2015	0.041	0.055	[−0.066, 0.149]	0.452
Prevalence	2005	0.016	0.067	[−0.115, 0.146]	0.815
	2010	0.039	0.035	[−0.030, 0.108]	0.266
	2015	0.037	0.053	[−0.068, 0.141]	0.489
Mortality	2005	−0.004	0.054	[−0.110, 0.102]	0.941
	2010	0.037	0.022	[−0.005, 0.080]	0.084
	2015	0.046	0.053	[−0.059, 0.150]	0.389

Notes. Sensitivity analyses were conducted to evaluate the linearity assumption underlying the year-specific ordinary least-squares (OLS) models. For each exposure year and dementia outcome, the fully adjusted model was re-estimated by including both the linear and quadratic terms for z-standardized per-capita food availability. Models additionally included all covariates listed in Table S1. All variables were z-standardized within the corresponding year-specific analytical sample, and heteroskedasticity-robust standard errors (HC3) were applied. The table reports estimates for the quadratic term only. No statistically significant quadratic effects were observed.

Table S8

Standardized OLS regressions with heteroskedasticity-robust (HC3) standard errors, restricted to countries with complete data across all exposure years and covariates (N = 92).

Outcome	Exposure year	β	HC3 SE	p-value	N
Incidence	2005	0.106	0.059	0.071	92
	2010	0.093	0.042	0.028	92
	2015	0.065	0.043	0.129	92
Prevalence	2005	0.105	0.057	0.067	92
	2010	0.094	0.043	0.028	92
	2015	0.063	0.043	0.144	92
Mortality	2005	−0.000	0.053	0.999	92
	2010	0.036	0.043	0.406	92
	2015	−0.004	0.028	0.878	92

Notes. This balanced-sample sensitivity analysis was conducted using the same set of countries with complete data across all exposure years (2005, 2010, and 2015) and covariates (N = 92). Year-specific multivariable ordinary least-squares (OLS) models were estimated after z-standardizing all variables within each exposure year; therefore, regression coefficients (β) represent standardized effect sizes. Models included per-capita food availability as the primary exposure together with all covariates listed in Table S1. Heteroskedasticity-robust standard errors (HC3) were used throughout to account for potential heteroskedasticity. Restricting the analyses to an identical set of countries ensured that comparisons across exposure windows were not influenced by differences in sample composition.

Table S9

Correlations between per-capita food availability across exposure years (balanced sample, n = 92)

Exposure Years	Pearson r	p-value
**2005 – 2010**	**0.866**	**< 0.001**
**2010 – 2015**	**0.964**	**< 0.001**
**2005 – 2015**	**0.828**	**< 0.001**

Notes. Pearson correlation coefficients were computed using the balanced sample of countries with complete data across all exposure years (2005, 2010, and 2015) and covariates (N = 92). Correlations were calculated using year-specific per-capita food availability estimates. All correlations were statistically significant ($p < 0.001$), indicating substantial temporal stability of food availability across exposure windows.

Table S10

List of countries included in the balanced sample (n = 92)

Albania, Algeria, Angola, Antigua and Barbuda, Argentina, Australia, Austria, Bangladesh, Barbados, Benin, Brazil, Bulgaria, Burkina Faso, Cambodia, Cameroon, Canada, Central African Republic, Chad, China, Colombia, Congo, Cote d'Ivoire, Cuba, Denmark, Djibouti, Dominica, Dominican Republic, Egypt, Ethiopia, France, Gabon, Germany, Ghana, Greece, Grenada, Guinea, Guinea-Bissau, Hungary, Iceland, India, Indonesia, Iran, Iraq, Ireland, Japan, Jordan, Kenya, Kuwait, Laos, Lesotho, Madagascar, Malawi, Malaysia, Mali, Mauritania, Mauritius, Mexico, Mongolia, Morocco, Mozambique, Myanmar, Namibia, Netherlands, Niger, Nigeria, Norway, Pakistan, Paraguay, Peru, Philippines, Portugal, Romania, Rwanda, Saudi Arabia, Senegal, Slovakia, South Africa, Spain, Sri Lanka, Sweden, Switzerland, Tanzania, Thailand, The Gambia, Togo, Trinidad and Tobago, Tunisia, Ukraine, United Kingdom, United States, Vietnam, Zambia, Zimbabwe.

Notes: The balanced sample includes countries with complete data across all exposure windows (2005, 2010, 2015) and all covariates used in fully adjusted models.

Table S11. Sensitivity analysis using alternative covariate-adjustment sets

Standardized ordinary least-squares (OLS) regressions with heteroskedasticity-robust (HC3) standard errors.

Outcome	Exposure year	Adjustment set	β (Food)	HC3 SE	p-value	N
Incidence	2005	Food availability only	0.722	0.088	<0.001	92
		Structural	0.128	0.085	0.133	92
		Structural + behavioral	0.123	0.081	0.130	92
		Full	0.106	0.059	0.071	92
	2010	Food availability only	0.656	0.068	<0.001	105
		Structural	0.150	0.065	0.020	105
		Structural + behavioral	0.133	0.066	0.043	105
		Full	0.110	0.039	0.004	105
	2015	Food availability only	0.588	0.083	<0.001	106
		Structural	0.174	0.121	0.151	106
		Structural + behavioral	0.014	0.107	0.896	106
		Full	0.030	0.065	0.638	106
Prevalence	2005	Food availability only	0.721	0.088	<0.001	92
		Structural	0.128	0.086	0.135	92

		Structural + behavioral	0.123	0.082	0.136	92
		Full	0.105	0.057	0.067	92
	2010	Food availability only	0.654	0.068	<0.001	105
		Structural	0.148	0.065	0.023	105
		Structural + behavioral	0.128	0.066	0.051	105
		Full	0.106	0.038	0.006	105
	2015	Food availability only	0.587	0.082	<0.001	106
		Structural	0.173	0.120	0.151	106
		Structural + behavioral	0.011	0.106	0.920	106
		Full	0.027	0.063	0.670	106
Mortality	2005	Food availability only	0.663	0.096	<0.001	92
		Structural	−0.011	0.106	0.920	92
		Structural + behavioral	−0.017	0.105	0.872	92
		Full	−0.000	0.053	0.999	92
	2010	Food availability only	0.594	0.069	<0.001	105
		Structural	0.052	0.083	0.529	105
		Structural + behavioral	0.029	0.088	0.743	105

	Full	0.037	0.036	0.303	105
2015	Food availability only	0.517	0.083	<0.001	106
	Structural	0.079	0.146	0.589	106
	Structural + behavioral	−0.104	0.136	0.442	106
	Full	−0.059	0.058	0.311	106

Notes. Alternative covariate-adjustment specifications were examined as a sensitivity analysis to evaluate the extent to which the association between per-capita food availability and dementia outcomes depended on the composition of the adjustment set. The *food availability only* model included no covariates. The *structural* model adjusted for GDP per capita, life expectancy, population aged ≥65 years, urbanization, and air pollution. The *structural + behavioral* model additionally included high alcohol use and smoking. The *full* model further included high body-mass index and high fasting plasma glucose and therefore corresponds to the fully adjusted specification used in the primary year-specific analyses. All variables included in each model were z-standardized within the corresponding exposure year. Heteroskedasticity-robust standard errors (HC3) were used throughout. β represents the standardized regression coefficient for per-capita food availability. The same year-specific complete-case sample was retained across adjustment specifications (N = 92, 105, and 106 for 2005, 2010, and 2015, respectively), ensuring that changes in the estimated coefficient reflect covariate adjustment rather than changes in sample composition. These models are intended as sensitivity analyses of progressively conditioned ecological associations and should not be interpreted as formal mediation models or as identifying distinct causal estimands.

Table S12. Leave-one-country-out sensitivity analysis of the fully adjusted year-specific models

Outcome	Exposure year	Full-model β	LOCO β range	LOCO p-value range	p < 0.05, n/N	p < 0.0056, n/N
Incidence	2005	0.106	0.076–0.128	0.035–0.109	4/92	0/92
	2010	0.110	0.092–0.130	0.001–0.015	**105/105**	**87/105**
	2015	0.030	0.006–0.078	0.076–0.923	0/106	0/106
Prevalence	2005	0.105	0.077–0.126	0.030–0.106	4/92	0/92
	2010	0.106	0.089–0.125	0.001–0.018	**105/105**	**25/105**
	2015	0.027	0.004–0.072	0.096–0.950	0/106	0/106
Mortality	2005	−0.000	−0.018–0.035	0.411–1.000	0/92	0/92
	2010	0.037	0.013–0.048	0.166–0.628	0/105	0/105
	2015	−0.059	−0.068–−0.007	0.240–0.798	0/106	0/106

Notes. Leave-one-country-out (LOCO) sensitivity analyses were conducted for each fully adjusted year-specific OLS model by iteratively excluding one country and re-estimating the model on the remaining countries. All variables were re-standardized within each reduced sample at each iteration, and heteroskedasticity-robust standard errors (HC3) were used. *Full-model β* denotes the standardized coefficient for per-capita food availability obtained from the corresponding model including all countries available for that exposure year. *LOCO β range* and *LOCO p-value range* report the minimum and maximum estimates obtained across all country-deletion iterations. The *p < 0.05* column reports the number of LOCO iterations in which the food-availability coefficient met the conventional significance threshold. The final column additionally reports the number of iterations meeting the Bonferroni-adjusted threshold used in the primary analyses ($\alpha = 0.0056$). The number of iterations corresponds to the number of countries included in each year-specific complete-case model (92 in 2005, 105 in 2010, and 106 in 2015). The analysis evaluates sensitivity to individual countries and is not intended to provide country-specific effect estimates.

Table S13. Sensitivity of ridge distributed-lag coefficients to alternative regularization strengths

Standardized ridge coefficients estimated at the cross-validated λ and at alternative penalty strengths (0.1×λ and 10×λ). Bootstrap 95% uncertainty intervals are reported for coefficients estimated at the cross-validated λ.

Outcome	λ selected	Penalty	β2005	β2010	β2015
Incidence	1.1220	0.1×λ	0.086	−0.067	0.072
		selected λ	0.083 [−0.090, 0.172]	−0.045 [−0.225, 0.158]	0.053 [−0.107, 0.224]
		10×λ	0.072	0.002	0.015
Prevalence	1.2589	0.1×λ	0.086	−0.051	0.056
		selected λ	0.083 [−0.083, 0.171]	−0.033 [−0.222, 0.154]	0.040 [−0.093, 0.212]
		10×λ	0.071	0.009	0.012
Mortality	0.0001	0.1×λ	−0.045	0.014	0.010
		selected λ	−0.045 [−0.137, 0.014]	0.014 [−0.146, 0.151]	0.010 [−0.100, 0.178]
		10×λ	−0.045	0.014	0.010

Notes. Ridge-regularized distributed-lag models were estimated in the balanced sample of countries with complete data across all exposure years and covariates (N = 92). Per-capita food availability measures for 2005, 2010, and 2015 were entered simultaneously together with the covariates included in the ridge specification, and all predictors and outcomes were z-standardized prior to estimation. For each dementia outcome, the ridge penalty parameter (λ) was selected using 10-fold cross-validation over a logarithmically spaced grid of 161 candidate values ranging from 10^{-4} to 10^{4}. At the selected λ, 95% uncertainty intervals were estimated using non-parametric bootstrap resampling (1,000 replicates), with λ fixed at the value selected in the original cross-validation procedure. Sensitivity to regularization strength was additionally evaluated by re-estimating each model at 0.1× and 10× the cross-validated λ. The relative magnitude of exposure-year coefficients showed some sensitivity to regularization strength, particularly for incidence and prevalence, whereas mortality coefficients were

essentially unchanged. All bootstrap uncertainty intervals at the selected λ included zero. Given the strong correlations among exposure windows, these ridge estimates are interpreted descriptively and should not be considered evidence of independent or temporally specific exposure effects.

Table S14. Regression diagnostics for the fully adjusted year-specific OLS models

Exposure year	Outcome	N	Leverage threshold (2p/N)	Maximum leverage	Observations above leverage threshold, n	Cook's D threshold (4/N)	Maximum Cook's D	Observations above Cook's D threshold, n	Maximum |studentized residual|	Observations with |studentized residual| > 3, n
2005	Incidence	92	0.217	0.739	10	0.043	1.763	11	4.351	2
2005	Prevalence	92	0.217	0.739	10	0.043	1.746	11	4.284	2
2005	Mortality	92	0.217	0.739	10	0.043	8.078	13	7.167	2
2010	Incidence	105	0.190	0.749	9	0.038	1.242	12	3.894	3
2010	Prevalence	105	0.190	0.749	9	0.038	1.154	12	4.537	2
2010	Mortality	105	0.190	0.749	9	0.038	7.591	12	5.860	2
2015	Incidence	106	0.189	0.769	9	0.038	0.519	11	7.072	2
2015	Prevalence	106	0.189	0.769	9	0.038	0.522	11	6.629	2
2015	Mortality	106	0.189	0.769	9	0.038	1.127	4	10.633	2

Notes. Regression diagnostics were evaluated for each fully adjusted year-specific ordinary least-squares (OLS) model. All models included standardized per-capita food availability and the full set of covariates (GDP per capita, life expectancy, population

aged ≥65 years, urbanization, air pollution, high alcohol use, high body-mass index, high fasting plasma glucose, and smoking). Potentially influential observations were assessed using leverage, Cook's distance, and externally studentized residuals. Conventional diagnostic thresholds were defined as leverage > 2p/N, where p denotes the number of estimated regression parameters including the intercept (p = 10); Cook's D > 4/N; and |externally studentized residual| > 3. Several observations exceeded one or more diagnostic thresholds, indicating the presence of potentially influential country-level observations. These diagnostic flags were not interpreted as automatic criteria for observation exclusion. Instead, their potential impact on the exposure estimates was evaluated using complementary leave-one-country-out analyses (Supplementary Table S12), which indicated that the principal findings were not driven by any single country. HC3 heteroskedasticity-robust standard errors were retained throughout the primary OLS analyses to provide inference robust to heteroskedasticity and leverage.
